# Stability of Hepatitis E Virus After Drying on Different Surfaces

**DOI:** 10.1007/s12560-022-09510-7

**Published:** 2022-01-27

**Authors:** Alexander Wolff, Taras Günther, Reimar Johne

**Affiliations:** German Federal Institute for Risk Assessment, Max-Dohrn-Straße 8-10, 10589 Berlin, Germany

**Keywords:** Hepatitis E virus, Drying, Stability, Inactivation, Surfaces

## Abstract

The hepatitis E virus (HEV) causes acute and chronic hepatitis in humans. The zoonotic HEV genotype 3 is mainly transmitted by consumption of contaminated food produced from infected animals. However, transmission via contaminated surfaces has also to be considered. Here, the genotype 3c strain 47832c was dried on steel, wood, plastics and ceramics, stored at 23 °C or 3 °C for up to 8 weeks and remaining infectivity was titrated on cell culture. During the drying process, only a mean 0.2 log_10_ decrease of HEV infectivity was observed. At 23 °C, remaining infectious virus was detected until week 4 on most surfaces, but HEV was completely inactivated (> 4 log_10_ decrease) after 8 weeks. At 3 °C, HEV was detectable up to 8 weeks on most surfaces, with an average 2.3 log_10_ decrease. HEV showed the highest stability on plastics, which was lower on ceramics and steel, and lowest on wood. The addition of bovine serum albumin mimicking high protein load had only a slight stabilizing effect. In conclusion, HEV shows a high stability against drying and subsequent storage on different surfaces. Strict application of hygienic measures during food production is therefore crucial in order to prevent HEV persistence on surfaces and subsequent cross-contamination.

**Supplementary Information:**

The online version contains supplementary material available at 10.1007/s12560-022-09510-7.

## Introduction

The hepatitis E virus (HEV) is an important agent of human hepatitis. Large outbreaks of hepatitis E were reported from developing countries, whereas sporadic cases are predominant in industrialized countries (Goel & Aggarwal, 2020; Webb & Dalton, 2019). In the last years, increasing numbers of hepatitis E cases have been notified in Europe (Aspinall et al., 2017). The disease is mainly characterized by acute hepatitis. However, chronic HEV infections, which can lead to life-threatening liver cirrhosis, are increasingly described in immunosuppressed transplant patients (Narayanan et al., 2019). In addition, extrahepatic manifestations like neurologic disorders have been attributed to HEV infection (Velavan et al., 2021).

HEV belongs to the family *Hepeviridae*, which is characterized by small single-stranded RNA viruses (Purdy et al., 2017). Two different particle forms are known for HEV: non-enveloped particles with a diameter of ~ 30 nm and quasi-enveloped particles with a diameter of ~ 40 nm (Nagashima et al., 2017). Whereas non-enveloped particles are mainly found in feces, quasi-enveloped particles exist in serum and cell culture supernatant (Wolff et al., 2020a; Yin et al., 2016a). Both particle forms are infectious in cell culture, with a higher infectivity of the non-enveloped particles compared to the enveloped particles (Capelli et al., 2020; Yin et al., 2016b).

Most of the human-pathogenic HEV strains are grouped into genotypes HEV-1 to HEV-4. HEV-1 and HEV-2 exclusively infect humans and their major route of transmission is via fecally contaminated drinking water (Pallerla et al., 2020). In contrast, HEV-3 and HEV-4 are zoonotic and circulate in reservoir animals such as wild boars and pigs (Pavio et al., 2017). The main transmission route of these genotypes is foodborne by consumption of undercooked meat or raw meat products from infected animals. Especially, raw liver and sausages containing raw liver have been linked to hepatitis E outbreaks in the past (Colson et al., 2010; Masuda et al., 2005; Matsuda et al., 2003). Furthermore, RNA of HEV was frequently detected in different meat products derived from domestic pigs, wild boars or deer (Pavio et al., 2017; Szabo et al., 2015).

The distinct stability of HEV in meat and meat products is mainly unknown because no reliable method for direct infectivity measurement of HEV in these matrices exists so far (Cook et al., 2017). Cell culture systems assessing the infectivity of HEV in solutions have been improved recently (Capelli et al., 2020; Meister et al., 2019; Schemmerer et al., 2016, 2019; Todt et al., 2020), but are still laborious and time-consuming compared to many other viruses. Recent stability studies using the cell culture-adapted HEV strain 47832c in phosphate-buffered saline (PBS) showed a very high resistance of HEV against a broad pH range and high salt concentrations (Wolff et al., 2020a, 2020b). Conditions prevailing in raw sausage production could not completely inactivate HEV, indicating that infectious virus can be expected in raw sausages if sufficiently contaminated starting material was used (Wolff et al., 2020a, 2020b). Another scenario of food contamination could involve cross-contamination via surfaces used during food production and preparation. However, no data on stability of HEV after drying on surfaces are available so far, making an assessment of the risk of HEV transmission through this pathway difficult.

In this study, the stability of HEV after drying on different surfaces was assessed, under conditions simulating those at food production and storage. Using a cell culture-based system for titration of HEV infectivity, the decrease of infectivity was analyzed directly after drying and after storage at 23 °C or 3 °C for up to 8 weeks. The results should help to estimate the risk of HEV transmission by cross-contamination during food production and through contaminated surfaces in general.

## Materials and Methods

### Virus and Cells

For all experiments, the HEV genotype 3c strain 47832c (GenBank acc. no. KC618403) was used. This strain was originally isolated from a serum sample of a chronically infected transplant patient (Johne et al., 2014). For production of a virus stock, an A549 cell line persistently infected with this virus strain (Johne et al., 2014, 2016) was cultivated. For infectivity titrations, the cell line A549/D3, a subclone of cell line A549 showing enhanced susceptibility to HEV strain 47832c (Johne et al., 2016; Schemmerer et al., 2016), was used.

### Preparation of Virus Stock

Virus stock preparation was performed as described (Wolff et al., 2020a). Briefly, the persistently HEV-infected A549 cell line was cultivated in a humidified incubator for 7 days and thereafter split 1:2 for culture expansion. For virus harvest, cells were subjected to a triple freeze/thaw cycle and the supernatant was collected and stored at − 20 °C until further use. Thereafter, the harvested supernatants were pooled, cell debris were removed by low-speed centrifugation and virus particles were concentrated via ultracentrifugation (Wolff et al., 2020a). The resulting pellets were redispersed in phosphate-buffered saline (PBS, PAN-Biotech GmbH, Germany) with 1/10 or 1/100 volume (depending on initial virus concentration) as compared to the original supernatant volume. After an additional cleaning step by centrifugation, the virus concentrates were combined, mixed, aliquoted and stored at − 20 °C. The resulting HEV stock dispersion had an infectivity of 2.8 × 10^4^ focus-forming units (ffu)/ml.

### Virus Stability Testing After Drying on Surfaces

A stock solution of bovine serum albumin (BSA), was prepared by dissolving 1.65 g BSA (Cell Signaling Technology, USA) in sterile PBS up to a total volume of 50 ml. The BSA solution was thereafter sterile filtrated (PES membrane, 0.22 µm, Merck Millipore Ltd., USA), aliquoted and stored at − 20 °C. To prepare an HEV stock preparation with BSA, 4 ml of the HEV stock dispersion was mixed with 0.4 ml of the BSA stock solution, leading to a final BSA concentration of 3 g/l, as suggested for virus inactivation studies on non-porous surfaces (Rabenau et al., 2012). To prepare a virus stock preparation without BSA, 4 ml of the HEV stock dispersion was mixed with 0.4 ml sterile PBS. The resulting virus titer of the preparations (with and without BSA) was 2.5 × 10^4^ ffu/ml.

Four different surface types were selected based on their common use in food production and during food preparation: steel (a common surface material in slaughter houses, cutting plants or groceries), wood and plastics (common material of cutting boards), and ceramics (commonly used for dishes). For contamination experiments, the sample carriers consisting of circular steel plates (stainless steel X2CrNi18-9, surface 2B, YC INOX CO., LTD., Taiwan), wood boards (European beech, Continenta GmbH, Germany), plastics boards (polyethylene, IKEA, Sweden) and spot plates of ceramics (glazed porcelain, Roth, Germany) were placed under a sterile workbench. Thereafter, 275 µl aliquots of the corresponding virus stock with or without BSA were placed on marked sites of the sample carrier of the corresponding material. Drying was done at room temperature. After 1 h, which resulted in a 2- to 3-fold volume reduction without complete drying, two aliquots were aspirated from each surface material with or without BSA using a pipette, put into a 1.5 ml tube and filled up to 0.5 ml with sterile PBS. Virus left on the surface was then picked up with a sterile PBS-moistened cotton swab (Boettger GmbH & Co. KG, Germany), which was placed into the tube containing the corresponding aspirated virus aliquots (sample “before drying”). Each tube was vortexed for 1 min, and the cotton swab was squeezed and removed from the tube. All tubes were centrifuged at 2000×*g* for 10 min at 4 °C and 450 µl of each supernatant was transferred into a new tube and stored at 4 °C until virus titration, which was performed at the same day. When the drying process was complete, the dried aliquots from each surface material were picked up with sterile PBS-moistened cotton swabs, placed into 1.5 ml tubes containing 0.5 ml sterile PBS and processed as described above (sample “after drying”, *t*_0_). For storage experiments, the sample carriers were removed from the bench after complete drying and immediately placed into a plastic box together with a data logger (OM-24, OMEGA, USA) recording temperature and relative humidity (RH) every hour. For the 23 °C experiment, the closed box was stored in the dark at room temperature. For the 3 °C experiment, two open water bowls (diameter: 20 cm) filled with aqua bidest. were placed within the box beside the sample carriers in order to maintain high humidity. After adding the data logger, the box was closed and stored in a dark cooling room. Samples were taken by swabbing as described above at 1 day, 1 week, 4 weeks and 8 weeks after contamination. All experiments were performed with two biological replicates.

### Titration of HEV Infectivity

HEV infectivity titrations were performed as described (Johne et al., 2016). Briefly, confluent A549/D3 cell layers were infected with tenfold dilution series of the samples in a 96-well plate format. Each of the two biological replicates derived from a specific experimental condition (see above) was titrated in four technical replicates, resulting in eight titrated subsamples for each experimental condition. After infection, the cells were incubated for 2 weeks and subsequently stained by immunofluorescence with an HEV capsid protein-specific rabbit antiserum and a fluorescein isothiocyanate (FITC)-conjugated secondary antibody. Fluorescent cell foci were manually counted using a confocal fluorescence microscope (Opera Phenix, PerkinElmer). A focus was defined as at least 2 contiguous cells showing a clear intraplasmatic fluorescence. The resulting foci numbers were multiplied by the dilution factors of the corresponding wells, in order to calculate ffu/ml, which were log_10_-transformed. Arithmetic means from the eight titrated subsamples of each distinct experimental condition and the respective standard deviations were calculated using MS Excel.

### Data Analysis

All three experiments were analyzed according to the differences between the conditions. As a prerequisite, all experimental results were tested for normal distribution using the Shapiro–Wilk test and *q*–*q* plots. As most results did not show a normal distribution, the Kruskal–Wallis test was used to analyze the general differences between all conditions for each experiment. In case of a significant result a pairwise comparison of the conditions was performed using the Wilcoxon test for unpaired samples, to identify respective significantly different pairs. For all statistical tests performed, a *p* value < 0.05 was defined as significant. All statistical tests were conducted with the software R 3.6.1 (R Core Team, 2020). For the descriptive analyses and the plots, the software MS Excel was used.

## Results

### Effect of Drying on HEV Infectivity

**Fig. 1:**
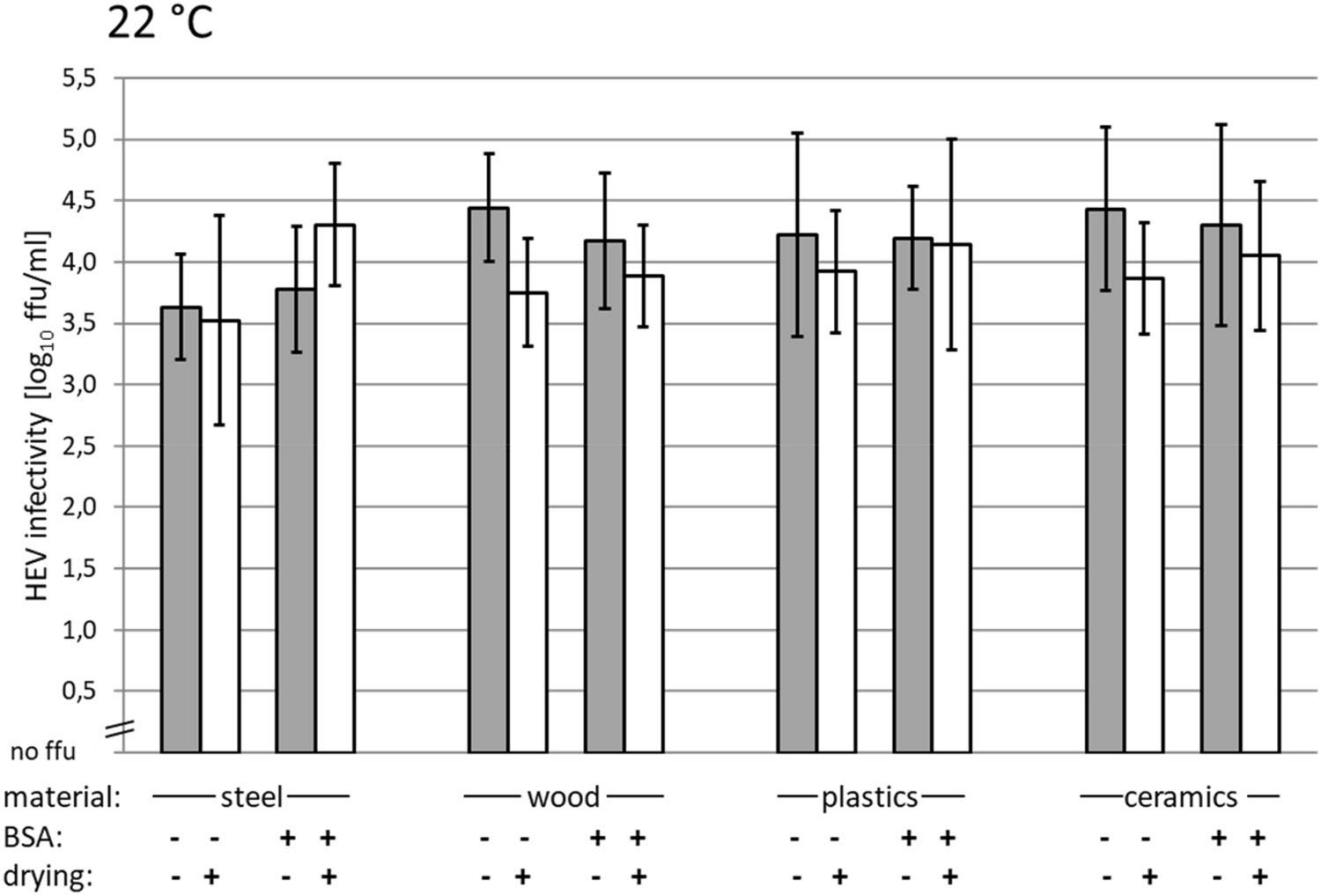
Stability of HEV against drying at 22 °C on different surfaces. The paired columns show infectivity before (gray) and after (white) drying. Each pair of columns represents a specific surface material in the presence or absence of bovine serum albumin (BSA) as loading substance. The arithmetic mean of two replicates, which were titrated in 4 replicates each, is shown. Scaled in log_10_ focus-forming units (ffu)/ml. Error bars indicate the standard deviations

The effect of drying on HEV infectivity was analyzed using four different surface materials and the presence or absence of BSA mimicking high protein load. HEV aliquots were added to the surface materials and the infectivity before and immediately after drying was assessed. The mean temperature during the drying process was 22 °C. As shown in Fig. 1, only minor differences in the mean HEV infectivity before and after drying are evident. In line with this, statistical analyses indicated no significant differences within all analyzed condition pairs. However, direct comparison of mean values might indicate a general trend of slightly decreased infectivity after drying.

In detail, the HEV mean infectivity titers decreased after drying for almost all conditions tested, in the range from 0.1 log_10_ ffu/ml for plastics with BSA to 0.7 log_10_ ffu/ml for wood without BSA. Only for steel with BSA, an increase in mean infectivity of 0.5 log_10_ ffu/ml was determined after drying; however, with overlapping standard deviations. The calculated arithmetic mean of the decrease after drying with and without BSA on all surfaces was 0.2 log_10_ ffu/ml.

By comparing the differences with and without BSA for each material, a general trend of slightly decreased infectivity without BSA as compared to the condition with BSA is likely. These mean differences range from 0.3 log_10_ ffu/ml for plastics and ceramics to 0.4 log_10_ ffu/ml for wood. Steel showed a mean difference of 0.6 log_10_ ffu/ml, including the mentioned increase of infectivity after drying with BSA.

By comparing different materials in case of the absence of BSA, steel showed the smallest inactivation effect with 0.1 log_10_ ffu/ml, followed by plastics with 0.3 log_10_ ffu/ml and ceramics with 0.6 log_10_ ffu/ml, whereas wood showed the strongest inactivation effect with 0.7 log_10_ ffu/ml. In the presence of BSA, steel showed the least inactivation effect of HEV with − 0.5 log_10_ ffu/ml, followed by plastics with 0.1 log_10_ ffu/ml, and the strongest inactivation effect was found by ceramics and wood with 0.3 log_10_ ffu/ml.

### Long-Term Stability of Dried HEV on Different Surfaces at 23 °C

**Fig. 2:**
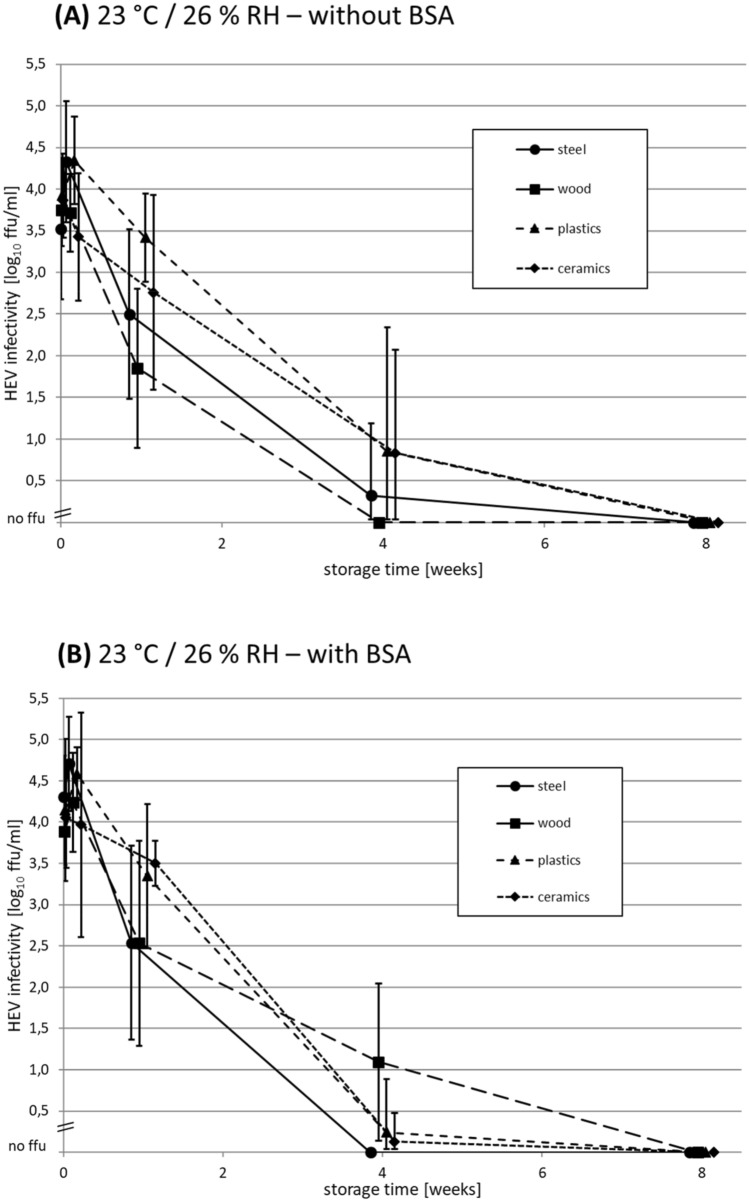
Time-course analysis of HEV infectivity after drying on different surface materials at 23 °C and 26% relative humidity (RH) for 8 weeks **A** without and **B** with adding of bovine serum albumin (BSA) as loading substance. Each data point represents the mean HEV infectivity on a specific surface material at the indicated time-point of storage. The arithmetic mean of two replicates, which were titrated in 4 replicates each, is shown. Scaled in log_10_ focus-forming units (ffu)/ml. Error bars indicate the standard deviations

In order to investigate the long-term stability of dried HEV at ambient conditions usually present in groceries or kitchens, a storage experiment for up to 8 weeks at room temperature and low RH was performed. Briefly, HEV with and without BSA was dried on four different surface materials and stored in a plastic box in the dark. The recorded data for temperature and RH are shown in Supplementary Data S1, indicating for the whole experiment arithmetic means of 23 °C and 26% RH. The results generated without BSA (Fig. 2A) and with BSA (Fig. 2B) indicate a continuous decline of infectivity, with minor differences between the surface materials.

Whereas the mean values at *t*_0_ (immediately after drying) and after one day storage were nearly identical, an almost linear decrease of mean infectivity was evident from 1 day to 4 weeks storage for all materials without BSA (Fig. 2A). The mean infectivity on wood declined to no infectivity (3.8 log_10_ ffu/ml decrease) after 4 weeks, whereas residual infectivity was detected at this time-point on the other surfaces, with mean infectivity decreases of 3.1 log_10_ ffu/ml for ceramics, 3.0 log_10_ ffu/ml for plastics and 3.2 log_10_ ffu/ml for steel. After 8 weeks, no residual infectivity could be detected for all materials.

The diagram in Fig. 2B, which shows the results for the experiment with BSA addition, is largely similar to that in Fig. 2A. Again, no residual infectivity was detected on all surfaces after 8 weeks. After 4 weeks, residual infectivities were determined on wood (2.8 log_10_ ffu/ml decrease), ceramics (4.0 log_10_ ffu/ml decrease) and plastics (3.9 log_10_ ffu/ml decrease), whereas no infectivity could be detected on steel at this time-point.

### Long-Term Stability of Dried HEV on Different Surfaces at 3 °C

**Fig. 3:**
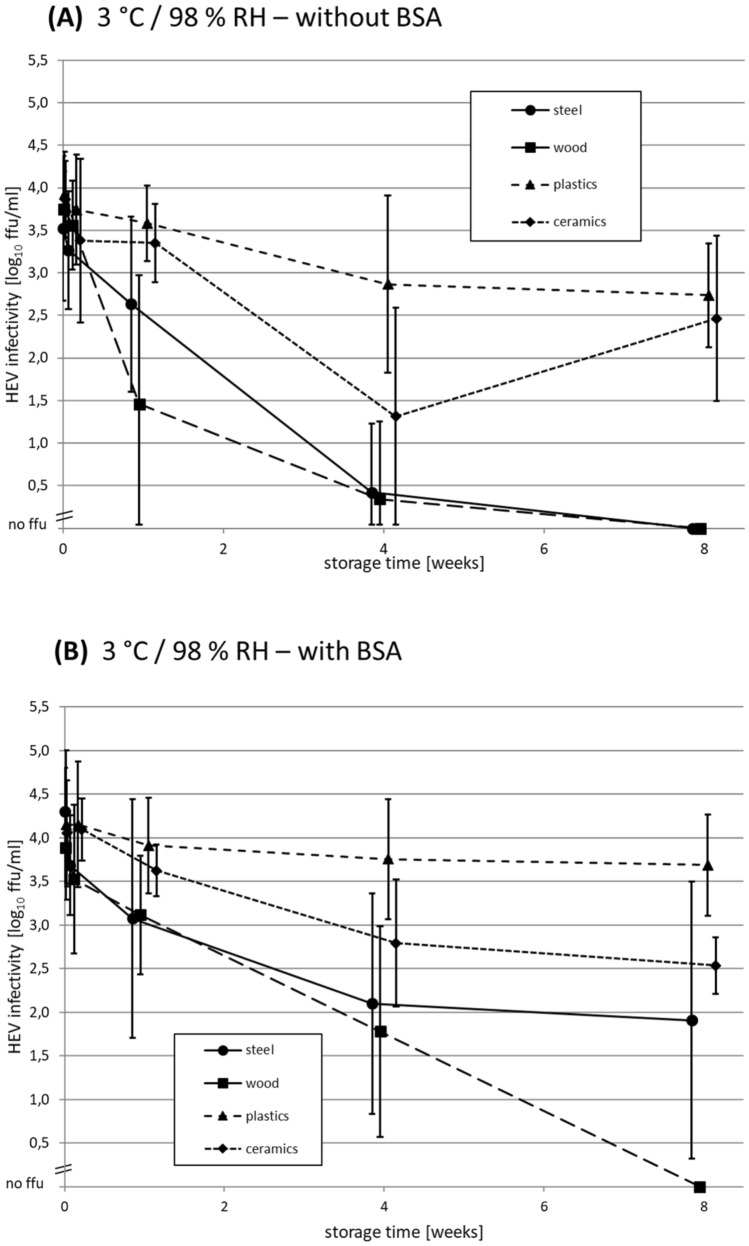
Time-course analysis of HEV infectivity after drying on different surface materials at 3 °C and 98% relative humidity (RH) for 8 weeks **A** without and **B** with adding of bovine serum albumin (BSA) as loading substance. Each data point represents the mean HEV infectivity on a specific surface material at the indicated time-point of storage. The arithmetic mean of two replicates, which were titrated in 4 replicates each, is shown. Scaled in log_10_ focus-forming units (ffu)/ml. Error bars indicate the standard deviations

In order to investigate the long-term stability of dried HEV at low temperature conditions usually present in refrigerators and cooling facilities, a storage experiment for 8 weeks in a cooling room at high RH was performed. Briefly, HEV with and without BSA was dried on four different surface materials and stored together with open water reservoirs in a plastic box placed in a cooling room in the dark. The recorded data for temperature and RH are shown in Supplementary Data S2, indicating for the whole experiment arithmetic means of 3 °C and 98% RH. After 40 days, the box with the samples had to be transferred to another cooling room, which resulted in a slight temperature increase of about 3 °C (Supplementary Data S2). The results generated without BSA (Fig. 3A) and with BSA (Fig. 3B) indicate a trend of a lower decline of infectivity as compared to the storage at 23 °C, with more obvious differences between the surface materials.

The data from the experiment without BSA (Fig. 3A) indicated similar mean infectivity values for *t*_0_ and one day of storage. Whereas for plastics only a slight mean decrease of about 1.0 log_10_ ffu/ml was observed within 4 weeks, the values for the other materials dropped more pronounced: for ceramics (2.6 log_10_ ffu/ml decrease), steel (3.1 log_10_ ffu/ml decrease) and wood (3.5 log_10_ ffu/ml decrease). Between weeks 4 and 8, the mean decrease of infectivity for plastics was minimal with 0.2 log_10_ ffu/ml, whereas no residual infectivity (> 3.5 log_10_ ffu/ml decrease) could be detected after 8 weeks storage for steel and wood. Ceramics showed a mean increase of infectivity of 1.2 log_10_ ffu/ml between 4 and 8 weeks; however, with large standard deviations especially at 4 weeks of storage. By comparing the inactivation curves shown in Fig. 3A, HEV shows a trend of the highest stability on plastics, followed by ceramics and steel, whereas HEV stability was lowest on wood.

By analyzing the data for the conditions with added BSA (Fig. 3B), no differences of mean infectivity between *t*_0_ and one day of storage was observed for plastics and ceramics, whereas a mean decrease of infectivity of about 0.5 log_10_ ffu/ml was detected for steel and wood. During storage for 8 weeks, HEV infectivity on plastics showed a mean decrease of only 0.4 log_10_ ffu/ml. At the same time, the mean HEV infectivity decreased on ceramics by 1.6 log_10_ ffu/ml, and on steel by 2.4 log_10_ ffu/ml. The largest mean decrease of infectivity showed HEV on wood with 3.9 log_10_ ffu/ml after 8 weeks of storage. The HEV inactivation curves shown in Fig. 3B indicate the trend that HEV was most stable on plastics, followed by ceramics and steel, and had the lowest stability on wood, under these conditions.

### Statistical Analyses

The result of statistical analysis for the first experiment (effect of drying) is described in chapter 3.1., indicating no significant differences between the analyzed conditions. The *p* values calculated from the storage experiments are summarized in Table 1.

**Table 1:** Statistical analyses of data from the experiments investigating storage of dried HEV on different surfaces

Conditions	*t*_0_ vs. 1 day	*t*_0_ vs. 1 week	*t*_0_ vs. 4 week	*t*_0_ vs. 8 week
Plastics, without BSA, 23 °C	0.183	0.570	**0.009**	**0.002**
Ceramics, without BSA, 23 °C	0.346	**0.037**	**0.004**	**0.002**
Steel, without BSA, 23 °C	0.167	0.115	**0.003**	**0.002**
Wood, without BSA, 23 °C	0.869	**0.003**	**0.002**	**0.002**
Plastics, with BSA, 23 °C	0.103	0.200	**0.002**	**0.002**
Ceramics, with BSA, 23 °C	0.776	0.126	**0.002**	**0.002**
Steel, with BSA, 23 °C	0.254	**0.007**	**0.002**	**0.002**
Wood, with BSA, 23 °C	0.223	**0.033**	**0.003**	**0.002**
Plastics, without BSA, 3 °C	0.803	0.296	0.058	**0.011**
Ceramics, without BSA, 3 °C	0.460	0.115	**0.004**	**0.004**
Steel, without BSA, 3 °C	0.734	0.083	**0.003**	**0.002**
Wood, without BSA, 3 °C	0.427	**0.008**	**0.002**	**0.002**
Plastics, with BSA, 3 °C	0.803	0.633	0.699	0.306
Ceramics, with BSA, 3 °C	1.000	0.234	**0.020**	**0.003**
Steel, with BSA, 3 °C	0.103	0.183	**0.004**	**0.006**
Wood, with BSA, 3 °C	0.356	0.059	**0.003**	**0.002**

Time point *t*_0_ was compared with all other time-points for all conditions. The calculation methods used are described in detail in chapter 2.5. Results are presented as *p* values. Bold *p* values indicate significant differences (*p* < 0.05)

As evident from Table 1, the frequency of significant differences between *t*_0_ and the other time-points (bold *p* values) increases with longer time intervals, indicating a time-dependent inactivation of HEV. In addition, more significant differences were found at 23 °C as compared to 3 °C, indicating faster inactivation at higher temperature. Only minor differences between the numbers of significant values derived with or without adding BSA are evident, indicating only a low effect of adding BSA. When comparing the surface materials, wood showed the highest number of significant differences between *t*_0_ and the other time-points, followed by steel and ceramics, whereas plastics showed the lowest number. This indicates that HEV inactivation is fastest on wood, followed by steel and ceramics, and slowest on plastics.

## Discussion

Knowledge about the stability of viruses under different environmental conditions is essential to uncover their transmission pathways, to develop concepts for prevention of virus transmission and to establish effective methods for virus inactivation. Although the assessment of HEV stability is still hampered by the lack of rapid and easy-to-perform methods for HEV infectivity determination, significant progress has been made in the evaluation of the stability of HEV against various physico-chemical treatments recently. Generally, a high stability of HEV against pH, salts, chlorine, UV light and high hydrostatic pressure was assessed in previous studies (Girones et al., 2014; Guerrero-Latorre et al., 2016; Imagawa et al., 2018; Johne et al., 2021; Wolff et al., 2020a, 2020b), which is in line with the major transmission pathways of HEV through contaminated food and water. In contrast, the stability of HEV after drying on surfaces is not known so far. However, contaminated surfaces may be involved in HEV transmission, e.g., by cross-contamination of food or by direct contact resulting in smear infections.

To investigate the HEV stability after drying, a cell culture system using the cell culture-adapted HEV subtype 3c strain 47832c was used (Johne et al., 2014). Subtype 3c represents one of the predominant subtypes detected in humans and animals in Europe (Adlhoch et al., 2016; Anheyer-Behmenburg et al., 2017). In addition, this cell culture system was used previously in several studies assessing the stability of strain 47832c (Johne et al., 2016, 2021; Wolff et al., 2020a, 2020b), enabling direct comparison of the data. In our study, the cell culture system enabled an initial assessment of HEV stability after drying on surfaces. However, as the method is still laborious and time-consuming, only a limited number of conditions and replicates could be investigated. Future studies using improved methods are therefore desirable in order to broaden the conditions to other surface materials and temperatures, and to gain more precise data regarding variation of mean errors.

In the tested preparation, both non-enveloped and quasi-enveloped particles are present (Wolff et al., 2020a). It has been shown that non-enveloped particles mainly occur in feces and quasi-enveloped particles in serum of patients, although non-enveloped HEV particles have also been identified in human sera in a recent study (Costafreda et al., 2021). The particle form present in pig liver, meat and meat products is unknown so far. However, the presence of a mixture of enveloped particles (budded from cells or originating from residual serum in the meat) and non-enveloped particles (released from damaged cells or generated by removal of the envelope by environmental factors) is most probable. Therefore, particle mixtures similar to that used in our experiments may reflect those occurring in meat in practice, although the distinct combination of particle forms in certain meat products may vary. A higher infectivity of non-enveloped particles as compared to quasi-enveloped particles has been described using cell culture studies (Capelli et al., 2020; Yin et al., 2016b), and a removal of the envelope due to environmental factors may therefore increase infectivity. In order to analyze the distinct contribution of inactivating and infectivity-increasing processes during drying and storage, additional experiments with preparations of separated non-enveloped and enveloped particles should be performed in future studies.

The resistance of viruses against the drying process represents the major factor for their survival in dried condition on surfaces (Sánchez & Bosch, 2016). In our experiments, only slight decreases of HEV infectivity have been found during the drying process, with no statistical significance between the titers before and after drying. This indicates that HEV is highly stable against drying, and the persistence of infectious virus has to be expected on surfaces after contact to contaminated meat or to excretions from infected animals or humans. The high stability was evident for all tested surface types and the addition of BSA, which was used as loading substance, had only a minor stabilizing effect. Other enterically transmitted viruses like hepatitis A virus (HAV), norovirus, rotavirus or astrovirus have also been shown to be highly stable against the drying process (Mahl & Sadler, 1975; Keswick et al., 1983; Sattar et al., 1986; Sobsey et al., 1988; Abad et al., 1994; Abad et al., 2001). By comparison of HAV, rotavirus, poliovirus and adenovirus during drying on different smooth surfaces, HAV showed the highest stability (Abad et al., 1994). According to the results of our study, HEV inactivation during drying showed similar characteristics as described for HAV. For example, in the absence of a loading substance during drying on ceramics, the mean infectivity was reduced by 0.5 log_10_ for HAV (Abad et al., 1994) and by 0.6 log_10_ for HEV. In the presence of a loading substance, the mean infectivity during drying on ceramics decreased by 0.5 log_10_ for HAV (Abad et al., 1994) and by 0.3 log_10_ for HEV.

The persistence of infectious HEV after drying on different surfaces and subsequent storage was assessed in our study at two different conditions, which simulated typical scenarios during food production and preparation. First, ambient conditions usually present in groceries or kitchens (or in hospitals, or generally in rooms) with a temperature of 23 °C and a low RH of 26% were tested. Second, low temperature conditions usually present in refrigerators and cooling facilities with a temperature of 3 °C and a high RH of 98% were chosen. This approach enabled the testing of typical scenarios, but the distinct contributions of temperature and RH to HEV inactivation could not be differentiated and should therefore be analyzed in future experiments. Generally, stability of HEV was lower at the ambient conditions compared to the low temperature conditions. Whereas at ambient conditions, HEV infectivity was mostly destroyed after 4 weeks and totally absent after 8 weeks, remaining infectious HEV could be detected at 8 weeks in 5/8 samples at low temperature conditions. Higher stability at 4 °C as compared to 20 °C has also been described for HAV, poliovirus and adenovirus after drying on surfaces (Abad et al., 1994), although the effect was less pronounced than for HEV.

Especially at the low temperature condition, a marked effect of the distinct surface material was observed in our study. Here, HEV showed an exceptionally high stability on plastics, with only 0.4 log_10_ ffu/ml infectivity decrease after 8 weeks in the experiment with addition of BSA. Ceramics and steel showed moderate inactivation rates, and HEV was almost completely inactivated (3.9 log_10_ ffu/ml decrease) on wood under the same conditions. The reasons for the differences are not known, but it could be speculated that the porous surface of wood absorbs water from the virus particles with higher efficiency as compared to non-porous surfaces, which may lead to a faster virus inactivation, as recently hypothesized for SARS-CoV-2 (Corpet, 2021). This is in line with results from experiments with HAV, showing a higher stability on smooth surfaces (aluminium, ceramics) as compared to porous surfaces (paper) (Abad et al., 1994). Generally, the stability of HEV and HAV at low temperature and high RH turns out to be rather similar, at least for surface materials where data are available for both viruses. For example, in the presence of a loading substance after drying on ceramics for 8 weeks, the mean infectivity was reduced by 1.1 log_10_ for HAV (Abad et al., 1994) and by 1.6 log_10_ for HEV, and on metal by 1.5 log_10_ for HAV (aluminium) (Abad et al., 1994) and by 2.4 log_10_ for HEV (steel).

Our study has some limitations. Because of the necessary use of the laborious and time-consuming titration system, only a low number of samples could be analyzed, thus limiting the analyzed conditions and replications of the experiments. As already mentioned, the distinct influence of temperature and humidity could not be differentiated because of the specifically selected experimental conditions. Also, a discrimination of the inactivation profile of non-enveloped vs. quasi-enveloped particles could not be assessed. In addition, only one strain has been investigated and other strains may show different behaviors. The use of PBS and addition of BSA may not completely reflect the complex composition of blood, feces or meat juice, which are suspected to be the most probable matrices containing HEV in the field. Generally, the transmission rate of HEV to surfaces and from the surfaces to food and humans has to be determined in the future to better assess the transmission probability under field conditions.

It can be concluded that HEV is highly resistant against the process of drying and shows a high stability against long-term storage on several surfaces. The highest stability was determined at low temperature and high RH resulting in detection of infectious virus for as long as 8 weeks in most cases. Therefore, remaining infectious virus has to be expected for long time on surfaces initially contaminated with HEV. Subsequent virus transmission to food by contact to the contaminated surfaces or direct virus transmission to humans by smear infections should therefore be considered. Although the distinct risk of human infection via these pathways cannot be assessed, because the minimal infectious dose for oral infection of humans is not known so far, preventive measures should be taken to minimize the transmission risk. This should include strict application of hygienic measures during food production to prevent cross-contamination of other food. Selection of suitable surface materials used during food preparation may also support hygienic measures as the type of surface material has been shown to have significant effects on HEV stability. Further studies should focus on testing of the HEV drying stability directly in biological matrices and include the analysis of transmission rates of HEV to and from surfaces to enable a more complete risk assessment on HEV transmission via surfaces.

## Supplementary Information

Below is the link to the electronic supplementary material.

Supplementary file1 (PPTX 108 kb)

## Data Availability

Data are available in Supplementary Materials and additional data can be retrieved upon request by R.J. (Reimar.Johne@bfr.bund.de).
