# Supplementary material for: Stability of Hepatitis E Virus After Drying on Different Surfaces
Source: Food Environ Virol. 2022 Jan 27;14(2):138–48. doi: 10.1007/s12560-022-09510-7 (PMC8793819; doi:10.1007/s12560-022-09510-7)
Supplement: Supplementary file 1 — Supplementary file1 (PPTX 108 kb) [file 12560_2022_9510_MOESM1_ESM.pptx]

## Slide 1
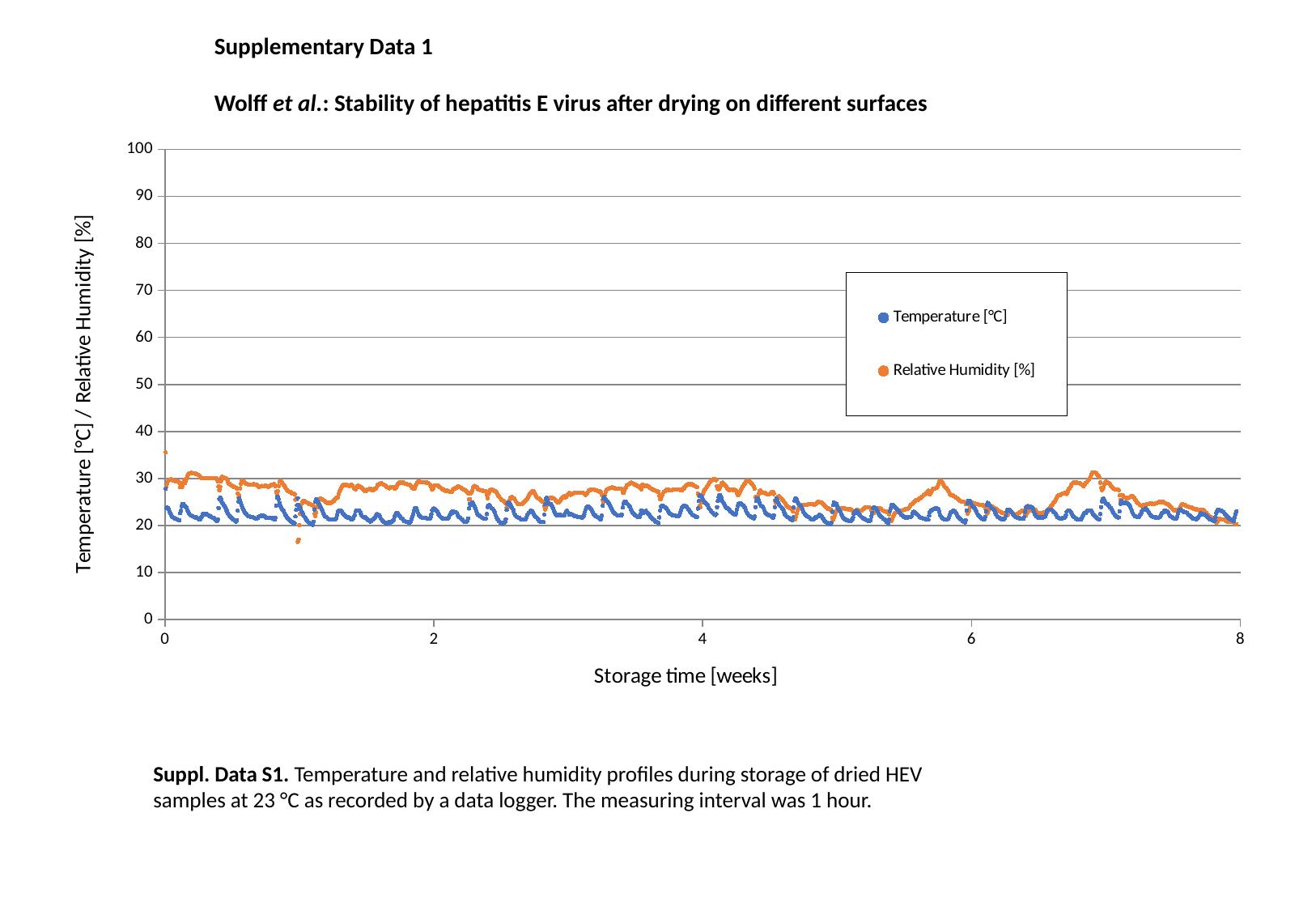

Supplementary Data 1
Wolff et al.: Stability of hepatitis E virus after drying on different surfaces
### Chart
| Category | Temperature [°C] | Relative Humidity [%] |
|---|---|---|Suppl. Data S1. Temperature and relative humidity profiles during storage of dried HEV samples at 23 °C as recorded by a data logger. The measuring interval was 1 hour.

## Slide 2
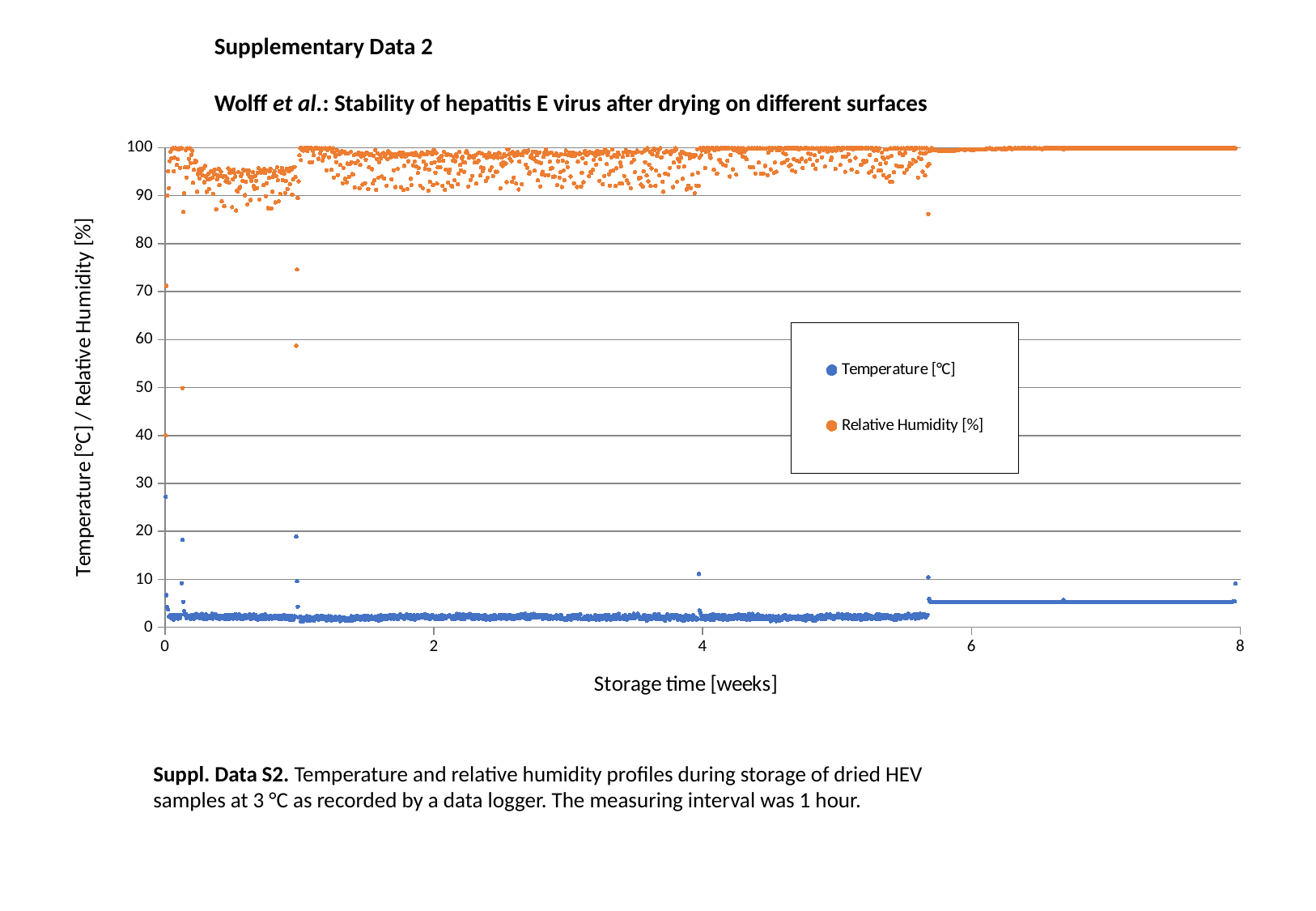

Supplementary Data 2
Wolff et al.: Stability of hepatitis E virus after drying on different surfaces
### Chart
| Category | Temperature [°C] | Relative Humidity [%] |
|---|---|---|Suppl. Data S2. Temperature and relative humidity profiles during storage of dried HEV samples at 3 °C as recorded by a data logger. The measuring interval was 1 hour.
